# The use of SGLT2 inhibitors in older people: What is important?

**DOI:** 10.1007/s40520-025-03156-8

**Published:** 2025-10-07

**Authors:** Giuseppe Armentaro, Raffaele Maio, Leonardo Bencivenga, Chukwuma Okoye, Giandomenico Severini, Velia Cassano, Valeria Zanobbi, Valentino Condoleo, Giuseppe Bellelli, Giuseppe Rengo, Andrea Ungar, Dario Leosco, Angela Sciacqua

**Affiliations:** 1 Geriatrics Division, “Renato Dulbecco” University Hospital of Catanzaro, Catanzaro, 88100 Italy; 2 Department of Translational Medical Sciences, University of Naples “Federico II”, Naples, Italy; 3 School of Medicine and Surgery, University of Milano-Bicocca, Monza, Italy; 4 Acute Geriatric Unit, IRCCS “San Gerardo dei Tintori”, Monza, Italy; 5 Department of Medical and Surgical Sciences, University Magna Graecia of Catanzaro, Catanzaro, 88100 Italy; 6 Department of Experimental and Clinical Medicine, University of Florence, Florence, Italy

**Keywords:** SGLT2i, Older patients, T2DM, Heart failure

## Abstract

Gliflozins, or sodium-glucose co-transporter 2 (SGLT2) inhibitors are synthetic derivatives of phlorizin. Phlorizin (phloretin-2-’O-β-glucopyranoside) is an O-glucoside of phloretin, a member of the dihydrochalcone family that is, in turn, a subclass of flavonoids. Isolated from the bark of the apple tree was initially used for its antipyretic and antimalarial effect. Many years later von Mering discovered its glycosuric properties.

SGLT2i, originally developed as oral hypoglycemic agents, have emerged as particularly valuable therapeutic options for older adults due to their efficacy and favourable safety profile, enabling glycaemic control without necessitating aggressive intervention strategies. Contemporary evidence demonstrates that SGLT2i maintains remarkable glycaemic efficacy across age stratifications, dispelling earlier concerns regarding diminished therapeutic response in older populations. The mechanism of action of SGLT2i, which involves inhibition of glucose reabsorption in the proximal renal tubules, remains functionally intact despite age-related physiological changes, including modest reductions in glomerular filtration rate commonly observed in geriatric patients. In this review the use of sglt2i in older patients will be analysed in detail.

## Graphical Abstract

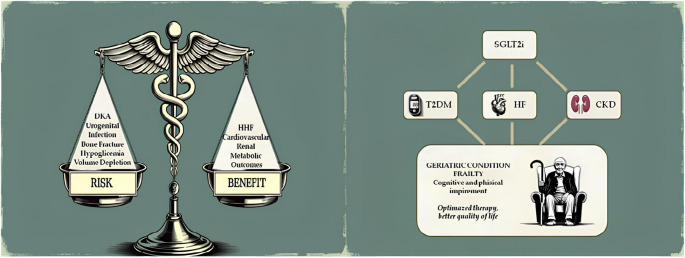

## Introduction: mechanism of action of SGLT2i

Gliflozins, or sodium-glucose co-transporter 2 (SGLT2) inhibitors are synthetic derivatives of phlorizin. Phlorizin (phloretin-2-’O-β-glucopyranoside) is an O-glucoside of phloretin, a member of the dihydrochalcone family that is, in turn, a subclass of flavonoids [1]. Isolated from the bark of the apple tree was initially used for its antipyretic and antimalarial effect [2–3]. Many years later von Mering discovered its glycosuric properties [4].

The pharmacological effect of phlorizines occurs through renal and intestinal blockade of sodium-glucose co-transporters 1 and 2 (SGLT1/2) and promotion of glucose excretion and improvement of insulin resistance. SGLT1/2 are transmembrane proteins that bind sodium to the extracellular surface. This bond involves the trapping of sugar molecules which, together with sodium, are transported inside from the outside of the cell [5]. This occurs by exploiting the electrochemical sodium gradient created by the sodium/potassium-ATPase which, by creating a gradient between the two sides of the cell membrane, allows SGLT1/2 to co-transport sodium and glucose from the extracellular side to the cytosol [6]. SGLTs are differently represented within the human body. SGLT1 is found predominantly in the small intestine where it plays an important role in passive water reabsorption [7]. SGLT2 is mainly localized in the first and second portions of the renal proximal tubule, where it co-transports a D-glucose molecule along with a sodium ion from the glomerular filtrate to the tubular epithelial cells. This activity is responsible for 90% of the reabsorption of filtered glucose. The remaining 10% is reabsorbed by SGLT1 in the third portion of the proximal tubule [8].

Based on the function of SGLTs have been developed, in the last decade, SGLT2 inhibitors (SGLT2i) as therapeutic agents to treat type-2 diabetes mellitus (T2DM). By inhibiting SGLT2, gliflozins reduce the renal threshold for glucose, leading to sustained glucosuria. This mechanism is insulin-independent, making it effective even in advanced diabetes with significant β-cell dysfunction. Daily urinary glucose excretion can reach 60–100 g, correlating with a blood glucose reduction of 20–25 mg/dL. Importantly, this process is accompanied by a mild osmotic diuresis and natriuresis, contributing to modest weight loss (2–3 kg) and blood pressure reduction (3–5 mmHg systolic), beneficial for obese and hypertensive patients.

SGLT2i includes drugs like empagliflozin, dapagliflozin, and canagliflozin that in large-scale trials (e.g., EMPA-REG OUTCOME, DAPA-HF) have revealed unexpected cardiorenal benefits beyond glucose lowering [9]. Chronic heart failure and kidney diseases are characterized by a significant inflammatory component that negatively influences their evolution. Furthermore, there is increasing evidence of how there is a close interaction between metabolism and inflammation and how their deregulation favors the development and progression of several pathologies; so much so that it is believed that the development of metabolic disorders can often be triggered or associated with inflammation [10–13].

One of the most relevant pleiotropic effects of SGLTi is linked to the anti-inflammatory properties that originate from the structure of their precursor, phlorizin. Recently, several studies have described the anti-inflammatory effect of SGLT2i using different experimental approaches. In particular SGLT2i have been proved to be able to modulate M1/M2 macrophage polarization and infiltration in different conditions. Macrophages are traditionally classified into two subgroups: M1, activated by Th1-type cytokines or bacterial lipopolysaccharides, in turn, produce pro-inflammatory cytokines, and M2 macrophages, which are activated by Th2-type cytokines and have anti-inflammatory effects [14].

The positive modulatory effect that SGLT2i on macrophage differentiation may explain, through the modulation of the inflammatory response, the positive effect shown by these drugs in many pathological conditions such as heart failure [15], diabetic cardiomyopathy [16] and diabetic nephropathy [17].

## Safety and efficacy of SGLT2i in clinical condition in older

### Safety and efficacy of SGLT2i in T2DM patients

The therapeutic management of type 2 diabetes mellitus (T2DM) in older populations presents considerable clinical complexities, due to multimorbidity, polypharmacy, and vulnerability to adverse drug reactions [18]. Sodium-glucose cotransporter-2 inhibitors (SGLT2i), originally developed as oral hypoglycemic agents, have emerged as particularly valuable therapeutic options for older adults due to their efficacy and favourable safety profile, enabling glycaemic control without necessitating aggressive intervention strategies [19–20].

#### Glycaemic efficacy in older populations

Contemporary evidence demonstrates that SGLT2i maintains remarkable glycaemic efficacy across age stratifications, dispelling earlier concerns regarding diminished therapeutic response in older populations. The mechanism of action of SGLT2i, which involves inhibition of glucose reabsorption in the proximal renal tubules, remains functionally intact despite age-related physiological changes, including modest reductions in glomerular filtration rate commonly observed in geriatric patients.

Post-hoc analyses from pivotal cardiovascular outcome trials have consistently revealed age-independent benefits. The CANVAS study subanalysis, encompassing over 4,300 participants aged ≥ 65 years, established that Canagliflozin demonstrated equivalent effectiveness in patients aged ≥ 75 years compared with younger cohorts, with mean HbA1c reductions of approximately 0.58% maintained across age groups [21].

Similarly, the EMPA-REG OUTCOME trial demonstrated consistent HbA1c reductions across all age groups with Empagliflozin, with older participants (≥ 65 years) achieving mean HbA1c reductions of 0.54% compared to placebo, virtually identical to younger populations [22]. Importantly, the time to achievement of glycaemic targets was comparable across age groups, suggesting that older patients do not experience delayed therapeutic onset. The DECLARE-TIMI 58 study further corroborated these findings, reporting that Dapagliflozin achieved HbA1c reductions of 0.6–0.8% in patients aged ≥ 65 years, with the upper range observed in treatment-naïve older patients [23]. Finally, the VERTIS CV study reporting that Ertugliflozin achieved HbA1c reductions of 0.70% with 5 mg and 0.72% with 15 mg in patients population with mean age 64.4 ± 8.1 years [24].

Recent systematic reviews and meta-analyses have provided additional validation of these age-independent effects with enhanced statistical precision. A comprehensive meta-analysis examining SGLT2i in frail or older patients with T2DM and heart failure found that whilst the magnitude of HbA1c reduction was modest in this population (0.4–0.5%), the consistency of glycaemic benefits across age groups was maintained, with no significant heterogeneity observed between age strata [25].

Notably, the glycaemic efficacy was preserved even in patients with multiple comorbidities and concurrent heart failure, conditions that frequently complicate diabetes management in older populations. The consistency of glycaemic response in older populations extends beyond HbA1c improvements to encompass broader metabolic parameters. Older patients demonstrate comparable reductions in fasting plasma glucose, with mean decreases ranging from 46 to 70 mg/dl across the major SGLT2i agents. Postprandial glucose excursions, which are often more pronounced in older patients due to age-related insulin resistance, show similar attenuation patterns to those observed in younger populations. The glycaemic benefits of SGLT2i in older patients appear to be independent of baseline renal function within clinically relevant ranges. Subgroup analyses have demonstrated maintained efficacy in patients with estimated glomerular filtration rates (eGFR) between 45 and 60 mL/min/1.73 m², a common finding in older diabetic populations. This characteristic distinguishes SGLT2i from several other antidiabetic agents that demonstrate diminished efficacy with declining renal function, making them particularly valuable in geriatric diabetes management where chronic kidney disease prevalence is elevated [21–25].

#### Safety profile and age-related considerations

Despite their established metabolic and cardio-renal benefits, SGLT2i presents specific safety considerations. The principal concerns include hypoglycaemic adverse events (HAEs), urinary tract infections (UTIs) and genital mycotic infections, volume depletion events, Diabetic Ketoacidosis and acute pancreatitis [26, 27].

#### Hypoglycaemic events

The risk profile for hypoglycaemia with SGLT2i in older patients appears favourable compared to other antidiabetic therapies. Sinclair et al. demonstrated that Canagliflozin use in individuals aged ≥ 75 years was associated with comparable HAEs rates in patients not receiving concurrent antihyperglycaemic agents and substantially lower hypoglycaemic risk compared with those treated with insulin or other glucose-lowering medications [28]. Pollack et al. demonstrated that dapagliflozin and empagliflozin exhibited a comparable incidence of HEA to that of the placebo group. However, the incidence of events is slightly higher in patients over 75 years of age. This result should be interpreted in light of the greater clinical complexity of elderly patients and their use of a greater number of hypoglycaemic drugs at risk of severe hypoglycaemia (es. sulphonylureas), a condition that often results from therapeutic inertia in optimising antidiabetic therapy in elderly patients [26]. Furthermore, Zhang et al. demonstrated in a recent meta-analysis that the incidence of HAEs did not differ between ertugliflozin and a comparator, irrespective of dosage [29]. Therefore, the risk of developing hypoglycaemia can be mitigated by means of drug therapy optimisation, reducing the risk of drug interactions and adverse reactions, through appropriate patient selection and through the provision of proper education to patients and their caregivers.

#### Genitourinary infections

The incidence of UTIs and genital mycotic infections (GMI) had a notable impact in SGLT2i therapy. Schorling et al. reported similar UTI rates between Empagliflozin and placebo across all age groups, with a notable sex-specific predisposition towards higher rates in females [30]. Importantly, treatment was demonstrated to be safe even in patients with recurrent UTIs, with approximately one-third of patients in both active treatment and placebo groups experiencing UTI events regardless of age. Cahn et al. similarly found no statistically significant increase in UTI or genital infections with Dapagliflozin, though numerically higher event rates were observed consistently across age groups [23]. Zhang et al. compared 10 clinical trials, evidence that Ertugliflozin versus placebo increased the risk of GMI and regardless of the dose, women were at greater risk of GMI than men compared with a comparator. However, treatment with ertugliflozin did not increase the risk of UTI compared with the comparators. These effects were irrespective of the daily dose [29].

#### Volume depletion

Volume depletion emerges as the most age-sensitive adverse effect associated with SGLT2i therapy. Multiple studies have demonstrated increased susceptibility in older populations, due to age-related physiological changes, concurrent diuretic use, and baseline cardiovascular comorbidities [23, 28, 30]. Schorling et al. identified higher volume depletion rates in patients aged 75–85 years, particularly those with baseline hypotension or concurrent antihypertensive therapy [30]. This finding underscores the importance of careful patient selection and monitoring volume status and blood pressure in older populations [31].

#### Diabetic ketoacidosis

The risk of diabetic ketoacidosis (DKA) with SGLT2i therapy appears consistent across age groups, with no age-specific increased susceptibility [23, 30]. However, the potential for atypical presentations of DKA in older patients necessitates heightened clinical vigilance [32].

#### Acute pancreatitis

Contradictory findings have emerged regarding the possible relationship between SGLT2i use and acute pancreatitis. However, no specific study has been conducted among the elderly population. A comprehensive analysis of the FDA Adverse Event Reporting System (FAERS) database demonstrated significant associations between SGLT2i treatment and acute pancreatitis. In the case of individual agents, canagliflozin demonstrated the strongest association with acute pancreatitis occurrence (Reported odd ratio (ROR) 5.03). Dapagliflozin and empagliflozin exhibited a comparable odd ratio (ROR 4.8), while ertugliflozin exhibited a lower odd ratio (ROR 3.58). It is intriguing to note that approximately 83% of adverse events transpired within a six-month period following the commencement of drug administration. It is interesting to observe that, of patients experiencing acute pancreatitis, 23.2% were aged 65–80 years and only 1.6% were over 80 years old [33].

When compared to other glucose-lowering drugs, SGLT2i demonstrates a relatively favorable pancreatic safety profile. A recent comparative analysis including 2,313 pancreatitis reports, indicates lower pancreatitis rates with SGLT2i compared to DPP-4 inhibitors and GLP-1 RA (14.7% vs. 15% and 70% respectively) [34].

Despite the emergence of evidence indicating a possible association between SGLT2i use and acute pancreatitis, the absolute risk remains low. Consequently, the clinical significance must be interpreted within the context of the well-established cardiometabolic benefits of the medication. The implementation of targeted pharmacovigilance research efforts will contribute to the further clarification of the risk-benefit profile of these important therapeutic agents in contemporary diabetes care.

#### Clinical implications and risk mitigation strategies

SGLT2i represents a clinically valuable therapeutic option for older patients with T2DM, offering consistent glycaemic efficacy whilst demonstrating long-term safety and tolerability across age groups. Successful implementation requires careful consideration of individual patient factors, including frailty status, comorbidity burden, and concurrent medication use. With appropriate patient selection, education and monitoring, SGLT2i can contribute meaningfully to the therapeutic armamentarium for T2DM management in older populations.

### Safety and efficacy of SGLT2 inhibitors in older adults with heart failure

Older adults represent the largest and most vulnerable group among individuals living with HF [35]. They often present with multimorbidity, frailty, and polypharmacy, all of which complicate therapeutic decisions and reduce adherence to guideline-directed therapies [36]. While guideline-based pharmacologic interventions have historically been underutilized in this population, recent evidence has confirmed that SGLT2 inhibitors are effective and generally well tolerated across the full spectrum of left ventricular ejection fraction, including in older and frail patients [20, 37–39].

#### Heart failure with reduced ejection fraction (HFrEF)

In patients with HFrEF, the landmark trials DAPA-HF and EMPEROR-Reduced enrolled substantial proportions of older adults (approximately 24% aged ≥ 75 years in DAPA-HF) [37, 38]. Subgroup analyses showed consistent benefits of dapagliflozin and empagliflozin in reducing the composite outcome of cardiovascular death and HF hospitalization across all age groups, without attenuation of efficacy in the older patient [20]. In addition to the reduction in cardiovascular death, SGLT2 inhibitors significantly lowered the risk of recurrent HF hospitalizations—a particularly meaningful outcome in older adults who are vulnerable to post-discharge decline and institutionalization [40]. Notably, improvements in health status scores such as the Kansas City Cardiomyopathy Questionnaire (KCCQ) were observed as early as 4 weeks after initiation, with sustained benefit [20]. In DAPA-HF, for instance, the hazard ratio for the primary outcome in patients ≥ 75 years was 0.68 (95% CI, 0.53–0.88), closely aligned with the overall trial population [37].

These benefits are particularly relevant in older patients, who experience higher baseline event rates and thus greater absolute risk reductions [39]. The early onset of benefit—typically within the first month—and the lack of need for titration render SGLT2i especially appealing in patients with limited functional or cognitive reserve [40]. Moreover, their favorable profile with regard to electrolyte balance, hypotension, and renal protection supports their use in the older, even in the context of polypharmacy [41].

#### Heart failure with mildly reduced ejection fraction (HFmrEF)

The intermediate phenotype of HFmrEF (left ventricular ejection fraction LVEF 41–49%) has long lacked targeted therapies. Both DELIVER and EMPEROR-Preserved included significant HFmrEF subgroups, and post-hoc analyses demonstrated that the efficacy of SGLT2i extends to this range of ejection fraction [42, 43]. Importantly, these trials included large numbers of older patients (mean age > 70 years), with consistent effects across age strata. In the DELIVER trial, the treatment effect of dapagliflozin was homogeneous in patients < 70, 70–79, and ≥ 80 years, with no increase in adverse events in the oldest group [42]. Furthermore, in older patients with HFmrEF, SGLT2 inhibitors reduced recurrent hospitalizations and preserved quality of life over time. In post-hoc analyses, patients ≥ 80 years derived comparable improvements in symptom burden and physical limitations, reinforcing their value in this under-recognized phenotype [44]. Improvements were observed not only in hard outcomes such as HF hospitalization, but also in quality of life metrics, including KCCQ scores [45]. These dimensions are especially meaningful in older adults, for whom functional independence and symptom relief are primary therapeutic goals.

#### Heart failure with preserved ejection fraction (HFpEF)

HFpEF is the most common form of HF in older adults, often overlapping with frailty, obesity, and chronic kidney disease [46]. Until recently, no pharmacologic therapy had convincingly improved outcomes in this phenotype. The EMPEROR-Preserved and DELIVER trials changed this paradigm, establishing SGLT2 inhibitors as the first drug class to improve outcomes in HFpEF [42, 43]. In EMPEROR-Preserved, approximately half of participants were aged ≥ 70 years, and empagliflozin reduced the composite of cardiovascular death and HF hospitalization consistently across age groups, including patients ≥ 80 [43]. DELIVER similarly showed preserved efficacy in the oldest patients, with numerically greater risk reductions in patients over 80 [42]. HFpEF remains the leading cause of HF hospitalization in older adults, often triggering functional decompensation. Both EMPEROR-Preserved and DELIVER showed reductions in total (first and recurrent) HF hospitalizations with SGLT2i. Moreover, significant gains were reported in patient-reported outcomes such as dyspnea relief, fatigue, and perceived functional capacity, particularly relevant in frail older patients [43–45].

These findings have been further supported by real-world cohort studies and meta-analyses showing that SGLT2i remains effective and safe in routine clinical practice, even among older and more frail populations [44, 47].

#### Safety profile in older adults

Despite age-related physiological changes, the overall safety profile of SGLT2 inhibitors in older adults is favorable. The most frequently reported adverse events include volume depletion, urinary tract infections, and transient declines in estimated glomerular filtration rate (eGFR). However, discontinuation rates due to adverse events have been low and similar to placebo in patients aged ≥ 75 or ≥ 80 years in major trials [20, 43, 47]. Importantly, SGLT2i has minimal interaction with other cardiovascular drugs and does not increase the risk of hyperkalemia or bradycardia, which is a major concern in older patients on RAAS inhibitors or beta-blockers [41]. Moreover, their fixed-dose administration and low monitoring burden make them ideal candidates for patients with limited adherence capacity or poor access to follow-up—both common in older populations.

### CKD and SGLT2is in older

Chronic kidney disease (CKD) is defined as a persistent eGFR < 60 mL/min/1.73 m2, and/or the presence of kidney damage, usually confirmed by the presence of albuminuria or an impaired albumin creatinine ratio (ACR) [48]. The prevalence of CKD in patients ≥ 65 years old is nearly 30% [49]. The mechanisms involved in the aging kidney are different and involve nephrosclerosis, interstitial fibrosis/tubule atrophy, arteriolosclerosis, and cellular senescence [50]. It represents a public health problem considering that reduced eGFR and albuminuria are associated with an increased risk of end stage renal disease (ESRD) and mortality [51] SGLT2is with their pleiotropic mechanism showed a renoprotective effect across the age and the levels of evidence, with safe side effects balance in older. The CREDENCE trial [52] is a RCT that included patients affected from T2DM and CKD, randomized to receive canagliflozin or placebo. The primary renal composite endpoint of ESRD, doubling of the serum creatinine level, or death from renal or cardiovascular causes, was reduced by 30% in the canagliflozin group. The renoprotective effects of canagliflozin was consistent in all the ages group [53] and a post hoc pooled analyses that stratified the CANVAS Program and CREDENCE patients according to frailty status revealed that adverse events were similar among frail and non-frail participants, except for osmotic diuresis [36]. The DAPA-CKD and EMPA-KIDNEY trails respectively focused on the effects of dapagliflozin and empagliflozin on the renal endpoint in both diabetic and non-diabetic patients affected by CKD. In the DAPA-CKD trial, dapagliflozin reduced the risk of the primary renal endpoint (a composite of sustained decline in the eGFR of at least 50%, ESRD, or death from renal or cardiovascular causes) of 44% compared to placebo. The protective effect of dapagliflozin was consistent in all the subgroups, reducing the relative risk both in patients aged ≤ 65 years (HR: 0.64, C.I.: 0.51–0.80) and > 65 years (HR: 0.58: C.I.: 0.48–0.88) [54]. A post-hoc analyses stratified the DAPA-CKD patients according to their frailty status measured by the Rockwood cumulative Frailty Index (FI), dividing the participants in not-to-mild (27%), moderately (38.2%), or severely frail (34.8%). Efficacy was assessed and dapagliflozin maintained a significant renoprotective role across the entire frailty spectrum compared to placebo. Safety analyses revealed that both in dapagliflozin that placebo group, greater level of frailty was associated with discontinuation from the study and increased serious adverse event (SAE). However, even if not significantly, SAE was lower in the dapagliflozin group than placebo. These data may suggest that frailty is a risk factor for adverse events, and a favourable benefit/risk profile arise for dapagliflozin use in patients with CKD and frailty [55]. In the EMPA-KIDNEY trial, empagliflozin reduced the risk of the primary renal endpoint (a composite of ESRD, a sustained decrease in eGFR to < 10 mL/min/1.73 m2, a sustained decrease in eGFR of ≥ 40% from baseline, or death from renal causes or death from cardiovascular causes) of 28% compared to placebo. The efficacy of empagliflozin was preserved in all ages, in both diabetic and non-diabetic patients, without significant difference in the adverse events [56]. In a post-hoc analyses of the EMPA-KIDNEY that used as indicator of frailty the predict risk of hospitalization, empagliflozin reduced the progression of renal disease or cardiovascular death irrespective of indicators of frailty, multimorbidity, or polypharmacy [57]. Beyond RCT, real world data confirm the efficacy and the risk/benefit balance of SGLT2is in older. In a retrospective study, Tumminia et al. included 364 patients aged over 65 years old and stratified in two groups according to the age superior or inferior 70 years old. The study found that in the younger group eGFR increased by 1.02 ml/min/year, while in the older group it declined by 0.42 ml/min/year. Discontinuation was similar across age groups, and side effects as genitourinary infections were the most frequent cause of treatment interruption in both study groups, while persistent eGFR decline and orthostatic hypotension were only present in older age class [58]. Similar results arise from a meta-analysis that included 13 study and 86,433 diabetic patients. Compared to the group of those aged < 65 years, the oldest group were associated with a higher risk of renal function impairment (OR 2.61) and volume depletion. Despite increased serious adverse events, most were mild, indicating an acceptable safety profile [59]. Taken together, this evidence confirms the renoprotective role of SGLT2is also in the geriatric patients, with benefits that overall exceed the risk of their use. Different mechanisms were proposed to explain the beneficial SGLT2is effects on kidneys. Furthermore, SGLTT2i can be successfully utilised even in cases of eGFR values as low as 20 ml/min/1.73 m2, thereby ensuring a substantial degree of cardio-renal protection in elderly patients suffering from CKD. Beyond the already known role in the haemodynamic of the glomerulus and the restore of the tubule-glomerular feedback, recent experimental models highlighted their anti-aging effects in the renal cells providing protection against ROS-induced cellular senescence, DNA damage, attenuating inflammation, reducing fibrosis and improving endothelial function, nominating SGLT2is as a new class of senolytic drugs [60].

### Other clinical conditions (SAS – COPD – aortic stenosis)

In addition to the known cardiovascular and non-cardiovascular benefits of SGLT2i, several studies have explored additional benefits of this drug class in other diseases; often as results of observational studies or sub-analyses of RCTs, thus not generalizable to the population, but nevertheless of interest.

In particular, the presence of sleep apnea syndrome, both obstructive and central, is a major comorbidity of HF older patients; and although several studies have evaluated various therapeutic approaches, none have been shown to improve their prognosis [61]. In this patient population, CPAP therapy represents the therapy of choice, but often burdened by reduced compliance. In this context, therapy with SGLT2i has been shown to improve polygraph parameters in patients with SAS with T2DM; and in patients with HF, T2DM, and SAS; specifically, by increasing the 60% probability of reducing Apnoea Hypopnea Hyndex by 50% after only 3 months of therapy, in the absence of CPAP therapy [62–64].

The promotion of weight loss, boosting lipolysis and reducing central adiposity, is one potential way that SGLT2i might help OSA patients to lessen OSA severity. Moreover, fat deposits around the neck and thorax may make upper airway collapsibility worse. Losing weight and reducing neck and thorax circumference may have a positive impact on airway collapsibility and AHI index. Indeed, the diuretic action of SGLT2i, through fluid reduction and redistribution may prevent nocturnal rostral fluid shift and lessen airway collapse reducing the severity of OSA [64].

#### Other clinical conditions – chronic obstructive pulmonary disease

Another pathology with a high prevalence in the older and diabetic population is chronic obstructive pulmonary disease (COPD); in this context, COPD exacerbations represent a major cause of morbidity and mortality [65]. COPD and T2DM share several pathophysiological mechanisms: systemic inflammation, oxidative stress, and metabolic dysfunction, which lead to accelerated respiratory decline and increased exacerbation frequency [65]. Therefore, in this context it seems reasonable to hypothesise a possible beneficial effect of SGLT2i. In particular, a cohort study compared the efficacy of SGLT2i vs. DPP4i on the risk of COPD exacerbations in a population with COPD and T2DM, and from this study it was found that SGLT2i therapy was associated with a 31% reduced risk of COPD exacerbations vs. DPP4i [66].

These results were confirmed by a recent meta-analysis conducted on 8 studies from registries or databases, evaluated the efficacy of SGLT2i compared to other anti-diabetic drugs, and showed that SGLT2i therapy was associated with a 35% reduction in the risk of exacerbations, compared to other anti-diabetic drugs [67].

#### Other clinical conditions – aortic stenosis

Aortic valvular stenosis (AS) is the most common valvular heart disease in the older population in industrialised countries. It is characterised by the progressive calcification and narrowing of the aortic valve, which results in a chronic pressure overload on the left ventricle, triggering a cascade of maladaptive myocardial remodelling that includes hypertrophy, interstitial fibrosis and, ultimately, heart failure [68]. The treatment of severe symptomatic forms of AS is transcatheter and surgical aortic valve replacement (TAVI/AVR), however clinical outcomes remain suboptimal in many patients, particularly those with advanced myocardial damage at the time of surgery [69]. Therefore, there appears to be a need to identify additional therapies capable of modifying the underlying pathophysiology and/or slowing the progression of AS from non-severe to severe forms. To investigate this hypothesis, an observational, multicentre, retrospective study evaluated the potential effect of SGLT2i therapy on the progression of non-severe to severe AS in a population of 1,698 patients, 458 of whom had been on SGLT2i for less than one year and 1,140 without SGLT2i. This study shows that SGLT2i therapy is associated with a 39% reduction in the risk of progression to severe AS (HR: 0.61; 95% CI: 0.39–0.94; *P* = 0.03), with a progressively lower risk in patients on SGLT2i for > 3, 6, and 12 months (HR: 0.54, 0.48, and 0.27, respectively) [70]. A possible pathophysiological explanation is the increased expression of SGLT2 at the myocardial level, which could accelerate cardiac remodelling; therefore, by acting at this level, SGLT2i could slow down cardiac fibrosis, inflammation, oxidative stress and metabolic dysfunction, slowing down the progression of AS [71]. Moreover, even in patients undergoing TAVI, SGLT2i therapy is associated with a better prognosis. In fact, the DapaTAVI (Dapagliflozin in Patients Undergoing Transcatheter Aortic-Valve Implantation) study showed that Dapagliflozin vs. placebo therapy was associated with a lower risk of death from all causes (HR 0.87; 95% CI, 0.59 to 1.28), and of Worsening of heart failure (subhazard ratio, 0.63; 95% CI, 0.45 to 0.88) [72].

#### Other clinical conditions – acute coronary syndrome

Ischaemic heart disease is a major cause of death in patients with atherosclerotic cardiovascular disease and T2DM. The EMPAREG-OUTCOME study had already been shown to reduce the risk of MACE in diabetic patients at high cardiovascular risk [22]. However, despite these assumptions, the use of empagliflozin or dapagliflozin in the EMMY, EMPACT-MI and DAPA-MI studies, conducted in patients with acute coronary syndrome, have not been shown to improve prognosis; therefore, it remains an open field of interest [73–75].

#### Other clinical conditions – cardio-oncology

Of particular interest in recent years, several studies have evaluated the potential impact of SGLT2i in the treatment of Cancer-Therapy-Related Cardiac Dysfunction (CTRCD). In particular, SGLT2i has been shown to improve symptoms and cardiac volumes in patients with anthracycline-induced CTRCD after 24 months of therapy [76].

A recent retrospective study included 1280 patients with a history of T2DM, cancer and exposure to potentially cardiotoxic antineoplastic therapies, who had cardiac dysfunction or HF related to cancer therapy (640 in each group, classified according to SGLT2i use). After a 2-year follow-up, patients taking SGLT2i in addition to conventional guided medical therapy had a lower risk of acute exacerbation of HF (OR: 0.483 [95% CI: 0.36–0.65); *p* < 0.001) and all-cause mortality (OR: 0.296 [95% CI: 0.22–0.40]; *p* = 0.001). The study confirmed the safety and efficacy of glyflozines in this patient category without new safety warnings, thus supporting the use of these drugs in active cancer patients [77].

There are many possible pathophysiological explanations: In mouse models, empagliflozin and dapagliflozin have been shown to preserve LVEF, reduce myocardial fibrosis, attenuate lipid peroxidation and inhibit caspase-3-dependent apoptosis in hearts exposed to doxorubicin; all of which result in improved mitochondrial bioenergetics, increased antioxidant defence systems (e.g. Nrf2/HO-1 pathway), modulation of autophagy and suppression of NLRP3 inflammasome activation; independent of glycaemic control [78]. Furthermore, through inhibition of the cardiac Na+/H + exchanger, empagliflozin increase ATP production in the myocardium and reduce cytosolic sodium and calcium overload, a mechanism implicated in doxorubicin-induced cardiotoxicity [79]. Therefore, SGLT2i represents an additional weapon to counteract cancer therapy-related cardiac dysfunction in humans.

## SGLT2i and geriatric syndromes

Following the repeated evidence of the beneficial effect of SGLT2i in the management of chronic diseases with high prevalence in older adults, discussed in the previous paragraphs, a growing interest has emerged in the scientific community for the potential impact of this pleiotropic therapeutic strategy in the management of the typical conditions of late life, in particular geriatric syndromes. Notwithstanding, specific research on the use of SGLT2i in these contexts is often based on observational studies or subanalyses of placebo-controlled clinical trials, thus limiting the generalizability of results to real-world clinical practice. Nonetheless, the prevalence of typical geriatric conditions such as sarcopenia, frailty and cognitive impairment, has increased over the decades, along with the aging of the population, therefore from the review of the literature it is possible to summarize some concepts related to SGLT2i employment, also with a view on future potential perspectives [80].

Frailty represents the most complex expression of biological aging, associated with negative outcomes in older adults, basically irrespective of the specific instrument used to assess it, among their heterogeneous multitude [81, 82].

Numerous chronic degenerative pathologies, involving the mechanisms of glucose homeostasis and macro/micro-vascular dynamics on which the benefits of this pleiotropic pharmacological class have been documented, contribute to the determination of variable states of vulnerability in older adults; accordingly, it is not surprising that SGLT2i could be considered as anti-frailty agent [83]. Nevertheless, as occurred for other clinical conditions, the presence of frailty determines underprescription of SGLT2i, due to adverse events concern, comorbidities, clinical complexity and limited chronological/biological age-specific evidence. It has been recently confirmed by a cross-sectional nationwide Danish study in patients with type 2 diabetes mellitus and cardiovascular disease: frailty was associated with a higher likelihood of forgoing the initiation of SGLT2i therapy [84]. Notably, the post hoc analyses of previously mentioned DELIVER and DAPA-HF trials, revealed that the benefits of dapagliflozin were independent on the frailty status, assessed through the Rockwood cumulative deficit model, even more pronounced as frailty increased [46, 85].

Indeed, the most robust evidence supporting the beneficial role of SGLT2i in frail people or those aged over 65 years comes from a meta-analysis of randomized clinical trials and observational studies involving 77,083 patients with diabetes mellitus and HF; Aldafas and colleagues reported that, although not significantly reducing HbA1c levels as well as macrovascular events, renal progression, acute kidney injury or diabetic ketoacidosis, SGLT2i were associated with lower risk of all-cause mortality, cardiac death, and hospitalisation for heart failure. Notably, the number-needed-to-treat decreased as frailty score increased, supporting increased efficacy of dapagliflozin in frailer participants (24). Not surprisingly, gliflozins efficacy was also detected in patients with HF stratified by frailty index, though to be verified with further data [86]. Accordingly, on the wake of previous evidence on the safety and efficacy of SGLT2i and Glucagon-Like Peptide-1 (GLP-1) receptor agonists in terms of cardiovascular outcomes and all-cause mortality, especially in frail older adults with diabetes mellitus [87], Hsiao and collaborators conducted a retrospective analysis comparing these two pharmacological classes in Taiwanese adults with type 2 diabetes mellitus. The authors confirmed the benefits on cardiovascular outcomes for both agents, even in frail individuals; interestingly, GLP-1 receptor agonists were associated with an increased risk of end-stage kidney impairment [88].

Although it constitutes a specific geriatric syndrome with a defined diagnostic pathway, sarcopenia represents a physiopathological substrate of general frailty that largely overlaps with physical frailty, significantly contributing to the risk of falls, fractures, and functional impairment up to disability [89, 90]. This condition has also been recognized as one of the most relevant complications of type 2 diabetes mellitus [91], therefore, in order to comprehensively evaluate the potential role of SGLT2is in the heterogeneity of the aging process, it is essential to consider their effect on muscle mass and function.

The Multidimensional Prognostic Index (MPI) is a tool that has been utilised in a variety of clinical contexts and across diverse cohorts of older adults afflicted with both acute and chronic diseases. It has consistently demonstrated a high degree of accuracy in the classification of populations based on their mortality risk and the occurrence of adverse health outcomes. The MPI provides a single numerical prognostic index that has the potential to facilitate clinical decision-making processes for the management of frail older adults [92].

Concerns emerged on the role exerted by SGLT2is on body composition, as body weight and fat mass are significantly reduced by gliflozins, but this effect, although beneficial for metabolic and cardiovascular control, seems to be paralleled by impairment in skeletal muscle mass [93].

As matter of the fact, findings from an important meta analysis comprising 25 randomized controlled trials accounting for 2,286 participants with type 2 diabetes mellitus has aroused much interest, since it reported SGLT2is to be related with beneficial results on fat mass loss and body mass reduction; contrarywise, detrimental effects were detected in muscle mass, in particular significant reduction in lean mass, skeletal muscle mass/index, and body water. Accordingly, authors warrant caution in patients already prone to physical frailty [94]. Afterwards, the Japanese EMPA-ELDERLY trial showed empagliflozin to determine significant decrease in body weight, body fat mass and water volume, with no compromission on muscle mass or strength, in older adults with type 2 diabetes mellitus [95]. Conversely, a post-marketing surveillance study retrieved a considerable number of cases of musculoskeletal and connective tissue disorder imputable to gliflozins [96]. Subsequent evidence has not helped to shed light on this complex relationship [97].

Closely related to sarcopenia and frailty, two further chapters particularly relevant in geriatric medicine deserve discussion in relation to the potential anti-aging properties of SGLT2is: falls and fractures. From the analysis of the literature, it emerges that the evidence is still limited and contrasting to be able to draw conclusions. On the other hand, already in 2019, a metanalysis was conducted to explore the effect of gliflozins on bone mineral density and fractures in people with type 2 diabetes mellitus, resulting in no significant association [98]. Interestingly, two research groups compared the risk of fractures between SGLT2i and dipeptidyl peptidase-4 (DPP-4) inhibitors. Cowan and colleagues conducted a cohort study including older adults with non-severe CKD [99], whereas a Korean research group focused on postmenopausal individuals aged ≥ 45 years [100]. Both findings suggest that SGLT2i use was not associated with an increased rate of incident fractures, even in populations at higher risk for fractures. In line with these results, a real-world insight on the safety of gliflozins was proposed through a European safety study by an Italian research group, which detected SGLT-2is to be associated with reduced reporting probability of falls, in comparison with DPP4is, consistent across sex and age groups [101].

Nevertheless, there is no shortage of studies that have demonstrated a negative effect on these outcomes. Especially, SGLT2i therapy determined an increased risk of vertebral fractures, compared to other antidiabetic agents, in Korean women with diabetes mellitus aged ≥ 65 years [102]. Similarly, the recently published results from a survey, conducted on totally autonomous patients with type 2 diabetes mellitus, revealed that SGLT2i use was an independent risk factor for falls, while no statistically significant association emerged for GLP-1 receptor agonists. Furthermore, the combined use of the two classes significantly increased falls risk [103].

Taken together, these reports suggest to accurately assess and manage this risk of falls and fractures when prescribing SGLT2i, waiting for further evidence to clarify the grey areas of this topic.

The progressive aging of the global population has also led to a relevant increase in the prevalence of cognitive disorders, including mild cognitive impairment (MCI) and dementia. Notably, T2DM represents a crucial risk factor for the onset and development of cognitive impairment, as it is associated with endothelial dysfunction, chronic inflammation, and metabolic alterations that can afflict brain function, thus explaining the great interest of the scientific community over time in the pleiotropic effects of pharmacological approaches to counteract glucose metabolism disorders [104]. Moreover, SGLT2 is expressed in the central nervous system and the blood–brain barrier, where it is involved in several processes: improved insulin sensitivity, modulation of inflammation, decreased apoptosis and oxidative stress, promotion of hippocampal synaptic plasticity [105]. Increased expression of SGLT2 has been reported in areas of human brain damage and within atherosclerotic plaques of diabetic patients [105, 106, 107]. Accordingly, gliflozins have emerged as useful agents for suppressing neuroinflammation, improving cerebral energy supply, modulating neurotrophic factors [e.g. Brain-Derived Neurotrophic Factor (BDNF), Nerve Growth Factor (NGF), and Glial Cell line-derived Neurotrophic Factor (GDNF)], enhancing synaptic plasticity, as well as potentially reducing the deposition and aggregation of amyloid-beta (Aβ) [105].

Preclinical models have been employed to evaluate the effects of SGLT2i on neuroinflammation, oxidative stress, cell death, and cognitive function, supporting the ability of this pharmacological agent to counteract microglial activation and the production of inflammatory cytokines, indicating protection against neuronal oxidative stress27. In the cortex and hippocampus of rats with Alzheimer’s disease and type 2 diabetes, empagliflozin reduced senile plaque density and Aβ levels [108]. In line with the demonstration of reduced neuroimmune damage provided by canagliflozin in cultured BV-2 microglia exposed to high glucose levels [109], a very recent paper reported the potential of dapagliflozin in alleviating neurodegeneration in rats model of cognitive impairment, through insulin-sensitizing, antioxidant activity and enhanced mitochondrial function [110]. These findings allow to speculate on protective effect against metabolically induced inflammatory toxicity.

Beyond experimental studies, several observational research and clinical trials focused on the role of gliflozins on neurodegenerative conditions [111]: regardless of the outcomes considered and the cognitive disorders examined, gliflozins proved to exert positive effect on cognitive function [112–115], or at least not deleterious [116]. In the context of the complexity of geriatric medicine, it is important to highlight the results from a perspective study on the intricate relationship between cognitive impairment and frailty, which showed the beneficial effect of 1 month therapy with empagliflozin on both Montreal Cognitive Assessment scores and 5-m gait speed test in frail older adults with diabetes and heart failure with preserved ejection fraction [117]. Furthermore, long-term therapy with gliflozins was associated with improved language domain and executive cognitive function in patients with type 2 diabetes mellitus [118]. The role of multidisciplinary approach was explored by a study in a Chinese population of older adults with MCI and type 2 diabetes mellitus: the authors demonstrated that the use of dapagliflozin combined with cognitive-behavioural training improved cognitive function and self-efficacy in metabolic control.

Summarizing, repeated intriguing evidence stimulate discussion around the potential neuroprotective properties of SGLT2is. Nevertheless, gaps in knowledge still remain, in particular exploring the long-term safety and effectiveness of gliflozins in the broad spectrum of neurodegenerative disorders, and the need for extending the research to non-diabetic cognitive impairment cases.

## In vivo and in vitro studies on SGLT2i – focus on hallmarks of aging

The sodium-glucose cotransporter 2 inhibitors (SGLT2i) have revolutionized diabetes management through their unique mechanism of promoting glucosuria via inhibition of renal glucose reabsorption (22). However, landmark cardiovascular outcome trials have revealed unexpected benefits extending far beyond glycemic control, including significant reductions in cardiovascular mortality, heart failure (HF) hospitalization, and progression of chronic kidney disease (CKD) [20, 119].

These pleiotropic effects have prompted investigation into the fundamental mechanisms underlying SGLT2i action, with particular attention to their potential impact on aging processes. The hallmarks of aging, as defined in 2013 and updated in 2023 by López-Otín et al. [120, 121], provide a comprehensive framework for understanding the molecular and cellular processes that drive aging and age-related pathology.

Genomic instability represents a fundamental driver of aging, characterized by the accumulation of DNA damage and chromosomal aberrations over time [122]. Several studies have investigated the potential genoprotective effects of SGLT2i.

In vitro studies conducted in human umbilical vein endothelial cells (HUVECs) exposed to high glucose conditions, demonstrated that SGLT2i significantly reduced DNA damage markers, including γ-H2AX foci formation and 8-oxoguanosine levels [123]. The protective effect was associated with enhanced activation of the DNA damage response pathway and improved DNA repair capacity. In vivo studies have corroborated these findings. In a study conducted in diabetic mice, chronic treatment with SGLT2i for 12 weeks resulted in reduced oxidative DNA damage in cardiac tissue, as evidenced by decreased 8-hydroxy-2’-deoxyguanosine (8-OHdG) levels and improved expression of DNA repair enzymes including OGG1 and PARP1 [124]. Similar protective effects have been observed in renal tissue, where SGLT2i treatment attenuated diabetes-induced DNA damage and preserved genomic integrity [125].

SGLT2i also has significant anti-inflammatory properties across multiple experimental systems. In vitro studies using immune cells stimulated with inflammatory mediators showed that SGLT2i treatment reduced pro-inflammatory cytokine production, including tumor necrosis factor α (TNF-α), interleukin-1β (IL-1β) and interleukin-6 (IL-6) [126]. The anti-inflammatory effects were mediated through inhibition of nuclear factor κB (NF-κB) signalling and activation of anti-inflammatory pathways including sirtuin 1 (SIRT1) and Nrf2.

Moreover, SGLT2i have been shown to influence epigenetic landscapes through multiple pathways. Epigenetic modifications accumulate with aging and contribute to altered gene expression patterns characteristic of senescent cells [127].

In diabetic cardiomyopathy models, SGLT2i treatment reversed pathological histone modifications, particularly H3K9me3 and H3K27me3 marks associated with gene silencing [128]. RNA sequencing analysis revealed restoration of youthful gene expression profiles, with particular enrichment in pathways related to mitochondrial biogenesis and antioxidant defence. DNA methylation studies conducted in aged mice treated with SGLT2i showed global hypomethylation reversal and specific demethylation of promoter regions of longevity-associated genes including Forkhead box O1 (FOXO1), SIRT1, and NRF2 [129]. These epigenetic changes correlated with improved metabolic function and extended lifespan in treated animals.

In addition, emerging evidence suggests that SGLT2i may influence telomere biology through multiple mechanisms. Telomere shortening serves as a molecular clock of cellular aging and has been implicated in various age-related pathologies.

In a clinical study involving patients with T2DM, treatment with SGLT2i for 6 months resulted in significantly longer leukocyte telomeres compared to standard care, accompanied by increased telomerase activity and upregulated expression of telomere-protective genes including Telomerase reverse transcriptase (TERT) and telomeric repeat binding factor 2 (TERF2) [130]. These effects were independent of glycaemic improvement, suggesting direct cellular protective mechanisms.

Mechanistic studies in cultured human endothelial cells revealed that SGLT2i treatment under hyperglycemic conditions prevented accelerated telomere shortening through reduction of oxidative stress and preservation of telomerase function [131]. The protective effect was mediated through activation of the SIRT1-FOXO3a signalling pathway, highlighting the interconnected nature of aging mechanisms.

SGLT2i influences multiple components of the nutrient sensing pathways, including Mechanistic target of rapamycin (mTOR), AMP-activated protein kinase (AMPK), and insulin/IGF-1 signalling, which play central roles in aging regulation.

In vitro studies demonstrate that SGLT2i activates AMPK independently of their glycosuric effects through alteration of cellular energy status [132]. This activation leads to downstream effects including enhanced autophagy, improved mitochondrial biogenesis, and activation of antioxidant pathways. The AMPK activation appears mediated through mild uncoupling of mitochondrial respiration, creating a beneficial metabolic stress response.

In addition, studies conducted in animal models demonstrated that SGLT2i treatment results in significant mTOR pathway modulation, with reduced mechanistic target of rapamycin complex 1 (mTORC1) activity in multiple tissues including heart, kidney, and liver [133]. This mTOR inhibition correlates with enhanced protein quality control mechanisms and improved cellular stress resistance.

Moreover, SGLT2i demonstrates significant mitochondrial protective effects across multiple experimental systems. Mitochondrial decline represents a central feature of aging, contributing to reduced cellular energetics and increased oxidative stress.

A study conducted in cultured cardiomyocytes exposed to hyperglycaemic conditions, demonstrated that treatment with SGLT2i preserved mitochondrial membrane potential, enhanced ATP production, and reduced mitochondrial reactive oxygen species (ROS) generation [134]. Mechanistic analysis revealed upregulation of peroxisome proliferator-activated receptor gamma coactivator 1 alpha (PGC-1α) and downstream mitochondrial biogenesis factors, along with improved mitochondrial quality control through enhanced mitophagy. In vivo studies conducted in diabetic animals consistently demonstrate mitochondrial protection with SGLT2i treatment. Cardiac tissue from dapagliflozin-treated mice showed increased mitochondrial density, improved respiratory complex activity, and enhanced antioxidant enzyme expression [135]. Similar benefits have been observed in renal and hepatic tissues, suggesting systemic mitochondrial protective effects.

Many studies also demonstrated that SGLT2i have significant anti-senescence properties through multiple mechanisms. In vitro senescence models using replicative exhaustion or stress-induced premature senescence, SGLT2i treatment reduced classical senescence markers including p21, p16INK4a, and senescence-associated β-galactosidase activity [136]. The anti-senescence effects were associated with reduced senescence-associated secretory phenotype (SASP) factor production and improved cellular stress resistance.

Animal studies in naturally aged mice showed that chronic SGLT2i treatment reduced tissue senescence burden, as evidenced by decreased p16INK4a-positive cells in multiple organs and reduced circulating SASP factors [137]. These effects correlated with improved physical function and extended health span in treated animals.

Recently, emerging evidence suggests SGLT2i may preserve stem cell function through multiple mechanisms.

In hematopoietic stem cell studies, SGLT2i treatment improved stem cell self-renewal capacity and differentiation potential in aged mice [138]. The protective effects were associated with reduced oxidative stress in the stem cell niche and preservation of stemness-associated gene expression profiles.

Cardiac stem cell studies demonstrated that SGLT2i treatment enhanced the regenerative potential of resident cardiac progenitor cells, with improved cell survival, proliferation, and differentiation capacity following ischemic injury [139]. These effects contributed to improved cardiac repair and functional recovery in treated animals.

The mechanisms underlying SGLT2i effects on aging hallmarks appear multifaceted and interconnected. Key pathways include: Metabolic reprogramming [140], Oxidative stress reduction [141], Autophagy enhancement [142], Sirtuins activation [143].

The accumulating evidence for SGLT2i effects on aging hallmarks has significant clinical implications. Beyond their established benefits in diabetes and cardiovascular disease, these agents may represent a novel approach to targeting fundamental aging processes.

The current evidence suggests that SGLT2i influences multiple hallmarks of aging through interconnected mechanisms involving metabolic reprogramming, stress response activation, and cellular protection. These effects may contribute significantly to the observed pleiotropic benefits of SGLT2i beyond glycaemic control. While much of the evidence comes from preclinical studies, the consistency of findings across multiple experimental systems and the correlation with clinical outcomes support the hypothesis that SGLT2i may function as genoprotective agents. Further research is needed to fully characterize these effects and translate them into clinical applications for healthy aging and age-related disease prevention.

## Data Availability

No datasets were generated or analysed during the current study.
